# The dynamics between in vitro culture and metabolism: embryonic adaptation to environmental changes

**DOI:** 10.1038/s41598-020-72221-1

**Published:** 2020-09-24

**Authors:** Camila Bruna de Lima, Érika Cristina dos Santos, Jéssica Ispada, Patrícia Kubo Fontes, Marcelo Fábio Gouveia Nogueira, Charles Morphy Dias dos Santos, Marcella Pecora Milazzotto

**Affiliations:** 1 Institute of Biomedical Sciences, University of São Paulo, São Paulo, Brazil; 2 Center for Natural and Human Sciences, Federal University of ABC, Av. dos Estados, 5001, Bairro Santa Terezinha, Bloco A, Lab 502-3, Santo André, SP CEP: 09210-580 Brazil; 3 Laboratory PhytoPharmaTech, Department of Pharmacology, Institute of Biosciences, São Paulo State University, Campus Botucatu, São Paulo, Brazil; 4 Department of Biological Sciences, School of Sciences and Languages, São Paulo State University, Campus Assis, São Paulo, Brazil

**Keywords:** Embryology, Energy metabolism

## Abstract

Previous studies have discussed the importance of an optimal range of metabolic activity during preimplantation development. To avoid factors than can trigger an undesirable trajectory, it is important to learn how nutrients and metabolites interact to help launching the correct developmental program of the embryo, and how much the in vitro culture system can impair this process. Here, using the bovine model, we describe a factorial experimental design used to investigate the biochemical and molecular signature of embryos in response to different combinations of morphological features—i.e. speed of development—and external stimuli during in vitro culture—i.e. different oxygen tensions and glucose supplementation. Our analyses demonstrate that the embryos present heterogeneous metabolic responses depending on early morphological phenotypes and the composition of their surroundings. However, despite the contribution of each single stimulus for the embryo phenotype, oxygen tension is determinant for such differences. The lower oxygen environment boosts the metabolism of embryos with faster kinetics, in particular those cultured in lower glucose concentrations.

## Introduction

Mammalian preimplantation development follows a generally accepted metabolic pattern. Yet, there is no consensus on how we can successfully use metabolism or metabolic biomarkers to predict embryos viability. According to the “quiet embryo” hypothesis^1^, embryo viability is associated with a relatively low metabolism, probably because viable cells spend less energy trying to repair genome and proteome damages. This proposal was further developed^2^ to introduce molecular components such as “the quiet range” of nutrients turnover, and then revisited many times to include different categories of “quietness” such as levels of intrinsic metabolic activity and loss of quietness due to environmental stress, particularly the oxidative stress often observed in culture systems that use high oxygen tension^1,2^. In 2011, Gardner^3^ suggested that, in human embryos, a significant increase in glucose consumption—indicating a highly active metabolism—is necessary during initial development to sustain embryo viability. Later on, Leese discussed the existence of an optimal range of metabolic activity, or, in other words, the embryo ability to successfully carry out the developmental program with the minimum amount of energy^4^.

The basis of our knowledge on preimplantation metabolism comes from studies on mouse and human embryos. When extrapolating to large animals, which have a better buffering capacity against metabolic insults^5^, certain precautions are required. Besides, in vitro based technologies for farm animals exhibit a number of side effects rarely described in other species^5^, reinforcing the importance of specific studies unraveling the interplay between in vitro culture and metabolism switches in the bovine model.

In the bovine developing embryo, prior to morulae compactation, the production of energy is based on the oxidation of pyruvate, which is preferred due to the low energy requirements at this point^6^. As embryonic development progresses, glucose consumption gradually increases. Oxidative metabolism has its first mark in the stage of 12–16 cells, when major genome activation occurs^6–8^. Within the cells, glucose follows different metabolic pathways, e.g., the pentose phosphate pathway, which generates both the ribose for DNA and RNA synthesis, and the NADPH for lipids biosynthesis and reduction of intracellular glutathione, a powerful antioxidant^6^. Internalized glucose can also be partially converted to lactate (known as the Warburg Effect), which, in vivo, could facilitate key events such as invasion, angiogenesis and modulation of the immune response during the implantation process^6^. This plasticity is particularly important for the initial embryo development given that highly proliferative cells have other metabolic requirements beyond the production of ATP. However, studies measuring simultaneously energy efficiency and nutrients uptake/production are still lacking.

Although it is largely accepted that carbohydrate metabolism (glucose, pyruvate and lactate) is crucial for embryonic development, the role played by the metabolism of endogenous lipids in this process remains poorly known. Lipids are a potential energy source for the embryo through the β-oxidation of fatty acids^9^. Such mechanism has been considered responsible for supporting the increase in energy demand for blastulation and hatching, besides contributing to the synthesis of biomass and favoring the redox system^10^. Numerous evidences show that embryos produced in vitro carry the marks of energetic stress, mainly due to inadequate support offered by existing culture systems^11^. Thus, providing conditions to foster metabolism, while avoiding, neutralizing or minimizing stress factors, may be the key to ensure proper development.

Likewise, the embryonic kinetics seem to be related to the embryos viability and ability to respond to stress. The timing to complete the first cell divisions, in particular from 2 to 5 cells^12^, is a robust predictor of blastocyst formation in humans^13,14^. In the bovine, the kinetics of the first 2 cell divisions is associated with distinct phenotypes at the blastocyst stage^15^. Molecular and biochemical characterization demonstrated that fast-developing embryos exhibit improved ability to trigger adaptive responses^15–17^ and distinct profiles of epigenetic reprogramming (more DNA methylation)^18^.

The scenario described above allows us to recognize the heterogeneity of embryonic metabolism and responses to external factors when fast and slow embryos are compared. Such heterogeneity should be considered as we explore some overarching questions:

(a) Is there a range to up/downregulate embryo metabolism while still sustaining its viability?

(b) How does heterogeneity affect metabolism and the ability to respond to environmental stressors?

(c) Can we produce embryo with an efficient energy metabolism using the available in vitro culture systems?

Avoidance of factors triggering undesirable trajectories should become easier as we learn about the influences of specific nutrients and metabolites on the developmental programming of the embryos^1^. Thus, in the present paper we investigate the biochemical and molecular signature of Fast and Slow bovine embryos in response to combined stressful stimuli during in vitro culture.

## Material and methods

### Experimental design

**Figure 1:**
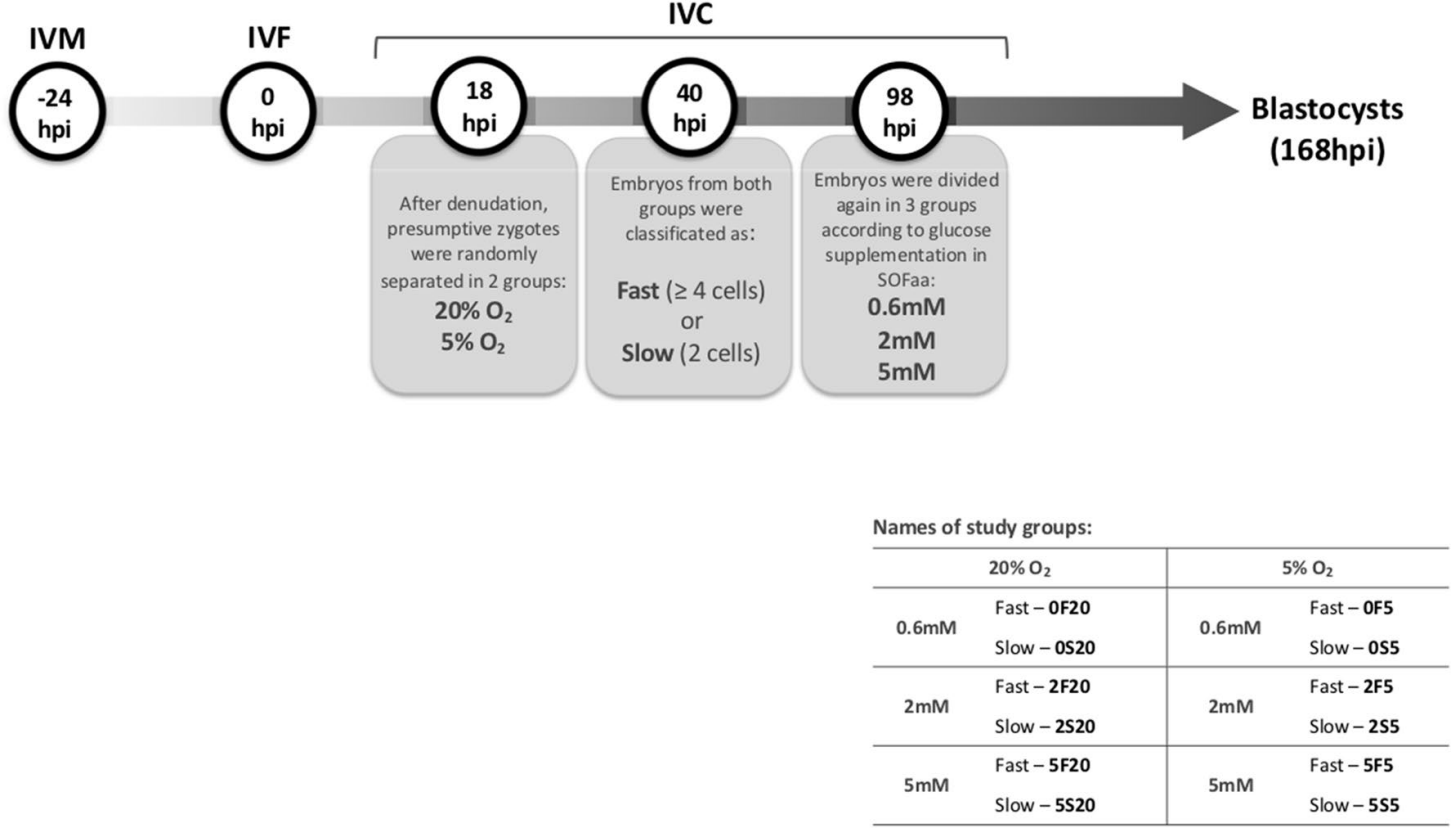
After in vitro maturation and fertilization, the presumed zygotes were randomly distributed for in vitro culture at 20 or 5% of oxygen and remained in the same oxygen tension for the total time of culture. At 40 h post insemination (hpi), the embryos were again assessed, classified and separated as Fast (≥ 4 cells) or Slow (2 or 3 cells). About 98 hpi, the embryos of each group were again randomly transferred to 3 glucose treatments (0.6, 2 or 5 mM) resulting in a total of 12 study groups.

Bovine embryos were in vitro produced following the experimental design as described on Fig. 1. All products were obtained from Sigma Aldrich, unless otherwise stated.

### In vitro production of bovine embryos (IVP)

Bovine ovaries were obtained from a commercial slaughterhouse and transported in warm sterile saline [0.9% (w/v) NaCl] for about 3 h. In the laboratory the ovaries were washed several times again with sterile saline to remove blood and debris. Cumulus-oocyte complexes (COCs) were collected by aspiration of follicles with diameter between 2 and 8 mm using syringe and needle.

For in vitro maturation (IVM), groups of 25–30 oocytes were placed in 90 μL drops of the maturation medium (TCM-199 bicarbonate supplemented with 10% of FBS, 0.5 μg/mL FSH [Folltropin-V, Bioniche, Belleville, Canada], 100 IU/mL hCG [Chorulon, Merck Animal Health, Boxmeer, The Netherlands]) under mineral oil. Maturation of COCs followed in an incubator at 38.5 °C, 5% CO_2_ in air and high humidity for 24 h.

Frozen commercial semen straws from 2 bulls were thawed in a water bath at 37 °C for 30 s and their contents were mixed and centrifuged on a discontinuous Percoll gradient (45% and 90%) as a strategy to avoid the influence of the bull in the kinetics of embryo development. Sperm concentration was adjusted to 1 × 10^6^ sperm/mL. Matured COCs were transferred to 90 μL drops of fertilization media^19^ and covered with mineral oil. Oocytes and sperm were incubated at the same conditions of air and temperature for 18 h. This study did not require direct handling of animals.

### In vitro culture of bovine embryos

Approximately 18 h post insemination (hpi), remaining granulosa cells were completely removed, presumptive zygotes were transferred in groups of 25–30 to 90 µL drops of Synthetic Oviductal Fluid (SOFaa; supplemented with 5% FBS [Gibco #16140-014], essential and non-essential amino acids) and distributed to the study groups following the experimental design (Fig. 1).

To prepare glucose supplementation, we first measured the concentration of glucose in the batch of FBS (~ 0.6 mM). Then, we calculated the necessary quantities to reach the final concentrations of 2 mM and 5 mM of our treatment. At 98 hpi, the embryos were reallocated to these treatments in smaller groups (15–20 per drop).

Finally, at 120 hpi, embryos were individually transferred to 20 μL drops of fresh SOFaa inside manually carved microwells, an adaptation from the well of the well system^20,21^, where they remained for the last 48 h of culture. Expanded blastocysts and their respective culture media were collected at 168 hpi and kept at − 80 °C for further evaluation.

This experimental design originated 12 study groups that were respectively named according to (i) the amount of glucose in the culture media (0.6 mM [0], 2 mM [2] or 5 mM [5]); (ii) the speed of first cleavages (Fast [F] or Slow [S]) and (iii) the oxygen tension in the culture system (20% O_2_ [20] or 5% O_2_ [5]). Final groups are: 0F20, 0S20, 2F20, 2S20, 5F20, 5S20, 0F5, 0S5, 2F5, 2S5, 5F5, 5S5 (Fig. 1).

### Embryo metabolism assessments

Quantitative evaluation of pyruvate and lactate in culture media of individually cultured bovine blastocysts.

The consumption of pyruvate was quantified in culture media collected at 168 hpi (n = 60, 5 replicates per group) using the EnzyChrom Pyruvate Assay (Bioassay systems, California, EUA). The production of lactate was quantified in the same culture media (n = 134; minimum of 10 per group) using EnzyChrom Lactate Assay Kit (Bioassay systems, California, EUA). Both evaluations are based on enzymatic reactions with fluorometric detection and were performed in accordance with the manufacturer’s instructions.

### Relative quantification of reactive oxygen species (ROS) and mitochondrial membrane potential (MMP)

Expanded blastocysts were collected at 168 hpi and analyzed by means of ROS content (n = 147; minimum of 5 replicates per group) with CellROX Green Reagent (CRG) (Thermo Fisher) and MMP (n = 163; minimum of 5 per group) with MitoTracker Red CMXRos (Thermo Fisher). Briefly, blastocysts from all groups were individually transferred to a 50 µL drop of PBS containing CRG (5 µM) + MitoTracker (0.05 µM) and incubated in the dark for 30 min in the same conditions of IVC. After incubation, embryos were washed in PBS, mounted on slides in glycerol drops and immediately evaluated under fluorescence microscopy (Leica Microsystems DM16000 B [MitoTracker: Ex/Em 538–617 nm, CRG: Ex/Em 495–519 nm]). Stained blastocysts were individually photographed at 40 × magnification using the Leica Application Suite (LAS) software (Leica Microsystems Brazil).

### Quantification of ATP in bovine blastocysts

Pools of 2 expanded blastocysts per group were collected at 168hpi and used in 4 replicates for the quantification of ATP using the ApoSENSOR ADP/ATP Ratio Bioluminescent Assay Kit (BioVision Inc., Milpitas, CA, USA). Briefly, after 5 min in Nucleotide Releasing Buffer, embryonic ATP was converted to light by the catalyzing activity of the enzyme luciferase (ATP Monitoring Enzyme). Luminescence intensity was measured, and a standard curve was used to convert the luminescence output to ATP concentration.

### Relative quantification of lipids in bovine blastocysts

Lipids were extracted from individual blastocysts (n = 90; minimal of 5 replicates per group) collected at 168hpi using the Bligh & Dyer method and subsequently analyzed by Multiple Reactions Monitoring Mass Spectrometry (MRM-Profiling) following the protocol described in^22^. We selected 3 classes of lipids to include in the present analysis: Free Fatty Acids (30 transitions, including C16:0 and C18:1), Triacylglycerols (99 transitions) and Cholesteryl esters (49 transitions). A total of 178 lipids were monitored and are described in Supplementary Table 1.

**Table 1:** Score system for metabolic efficiency of in vitro culture embryos.

Variable		Assigned score
Pyruvate consumption	High	0
	Intermediate	1
	Low	2
Mitochondrial activity	Low	0
	Intermediate	1
	High	2
	Very high	0
ROS production	High	0
	Intermediate	1
	Low	2
ATP production	Low	0
	Intermediate	1
	High	2
Lipid ratio—FFA/(TAG + cholesterol)	High	0
	Intermediate	1
	Low	2
Blastocyst rate	Low	0
	Intermediate	1
	High	2

### Analysis of transcription profile of target genes in bovine blastocysts

#### Total RNA extraction and cDNA synthesis

Pools of three blastocysts per group were collected at 168 hpi and used in 4 replicates. Gene expression analysis was performed using inventoried TaqMan assays (20 ×, Applied Biosystems), specific for Bos taurus. We verified the mRNA abundance of 84 target genes (Supplementary Table 2) according to functional categories specifically chosen to aid the investigation of the metabolic response in bovine embryos.

Briefly, we utilized PicoPure RNA Isolation Kit (Applied Biosystems, USA KIT0204) for total RNA extraction and the High-Capacity cDNA Reverse Transcription Kit (Applied Biosystems, USA #4368814) for cDNA synthesis. Both protocols were performed following the manufacturer’s instructions. Each RNA sample was quantified with NanoDrop (NanoDrop, Thermo Fisher Scientific, USA) and final amount was adjusted to 50 ng prior to reverse transcription.

### Pre-amplification and biomark HD system

Pre-amplification and qPCR were performed as previously described^23^, following the guidelines of the manufacturer (Fluidigm, USA). Briefly, samples were submitted to sequence-specific preamplification in a mix containing 1.25 µL assay mix (final concentration of 0.2× for each TaqMan assay), 2.5 µL TaqMan PreAmp Master Mix (Applied Biosystems) and 1.25 µL cDNA. The reactions were initiated at 95 °C for 10 min followed by denaturing at 95ºC for 15 s, annealing and amplification at 60ºC for 4 min for 14 cycles. The preamplified products were diluted sixfold prior to qPCR.

The 96 × 96 Dynamic Array Integrated Fluidic Circuits chip (Fluidigm) was loaded according to the manufacturer's protocol with each sample solution (2.25 µL diluted-preamplified cDNA, 2.5 µL of TaqMan Universal PCR Master Mix [Applied Biosystems] and 0.25 µL of Sample Loading Reagent [Fluidigm, USA]); and each assay solution (2.5 µL of 20 × TaqMan Gene Expression Assay [Applied Biosystems] and 2.5 µL of 2 × Assay Loading Reagent [Fluidigm, USA]). The qPCR thermal cycling was performed in the Biomark HD System (Fluidigm, USA) using the protocol TaqMan GE 96 × 96 Standard. All the analysis was performed in four biological replicates and Ct values were calculated from the system’s software (Biomark Real-time PCR Analysis, Fluidigm, USA).

### Statistical analysis

The experimental design proposed for this work originated 12 study groups as previously described. Thus, we opted for a more descriptive approach to analyze the resulting data. When necessary to show the significance of the results, GraphPad Prism 8.0 was used to verify normality and then compare groups by Student t test or ANOVA + Tukey, considering an alpha of 5%. Descriptive results are summarized on Supplementary table 3.

### Blastocyst rates

Total cleavage rates were obtained from the number of cleaved embryos divided by the total number of presumed zygotes (Supplementary Table 4). Likewise, the ratio of Fast and Slow embryos was calculated according to the formula (number of Fast or Slow embryos/number of presumed zygotes). Blastocyst rates were calculated from the number of total blastocysts (i.e. initial blastocysts + blastocysts + expanded blastocysts + hatched blastocysts) obtained on Days 7 and 8 on the number of Fast or Slow embryos.

### Biochemical evidences to assess blastocyst metabolism

The concentration of pyruvate and lactate in the culture medium, and the amount of ATP in the blastocysts were obtained with the aid of a standard curve. Results were expressed as means ± SEM. For the quantification of ROS and mitochondrial membrane potential, the images of each embryo were analyzed using ImageJ software (National Institutes of Health, USA) to set a threshold for the background subtraction; resulting values for integrated density data were used to calculate the fluorescence intensity.

### Relative quantification of lipids in the bovine blastocyst

The results extracted from the mass spectrometer were processed using a script especially constructed to obtain the absolute intensity of each ion. Then ion intensities from transitions of interest were recovered for all groups and normalized by the total ion current (TIC) to obtain relative ion abundances.

### Gene expression analysis

For the relative quantification of the transcripts of interest, we obtained ΔCt values for each sample, calculated from the difference between the Ct values (Cycle Threshold) of each target gene and the geometric mean Ct values of the genes selected by the software NormFinder^24^ as stable endogenous controls (GAPDH and PPIA). Gene expression results were expressed as ΔCt and then normalized (scale from 0 to 1) for the creation of heatmap graphs.

### Cluster analysis of biochemical and molecular evidences

Parsimony analysis is a method of classification that groups clusters hierarchically into discrete sets and subsets^25^, and can be used to organize any kind of comparative data from different species or specimens (e.g., morphological or ecological characters, genes, human languages and behavior, etc.). The basic requirement for a parsimony analysis is to translate the observed information into characters. In this study, we included 93 characters among molecular and biochemical evidences. More specifically, they are 84 genes (listed on Supplementary Table 2), 5 biochemical evidences (quantification of ROS, mitochondrial activity and ATP in the embryos; quantity of lactate and pyruvate in the culture media) and 5 lipid evidences (average intensity of triacylglycerol, cholesteryl esters, free fatty acids, palmitic acid and oleic acid). All characters were normalized (scale from 0 to 1) and pooled into a single data matrix. The matrix was analyzed under a parsimony algorithm, with 500 replicates and Tree-Bisection and Reconnection (TBR) for heuristic searches, using the Traditional Search option in the TNT software—Tree Analysis Using New Technologies^26^. All characters were considered additive and equally weighted. All trees were created using the iTol tool^27^. The clusters were discussed based on the resulting unrooted dendrograms. Finally, the respective nodes and the main attributes that contributed to each cluster were identified.

### Creation of score system

A score system was created to fill the gap between the descriptive results obtained by parsimony analysis and the need of a more qualitative approach to help define the best/worst culture conditions. For that matter, we relied on a combination of analytical rating, assigning grades based on literature reports for the selected parameters, as well as critical thinking over the results obtained with this experimental design.

Results of biochemical analyses (pyruvate consumption, mitochondrial activity, ROS production, ATP, relative lipid quantities), as well as blastocyst rates, were gathered to create a database containing information from a total of 585 blastocysts (or their respective culture media). For every analyzed character, we verified the distribution plot, and embryos were allocated into quartiles. The 1st or 4th quartiles, representing the extreme of phenotypes, were given a score of 0 or 2, as described on Table 1. The 2nd and 3rd quartiles were given a score of 1, except for mitochondrial activity. For lipid amounts, the variable used was the ratio between free fatty acids and the sum of cholesteryl esters and TAG, to indicate the direction of lipid metabolism (oxidation/storage). Scores were assigned individually for each embryo. Detailed information on the attribution of the scores can be found on supplementary table 5. The partial score of each study group was obtained by the sum of the score for each embryo divided by the total number of replicates per group. The assignment of the scores will be further discussed in the results section.

## Results

**Figure 2:**
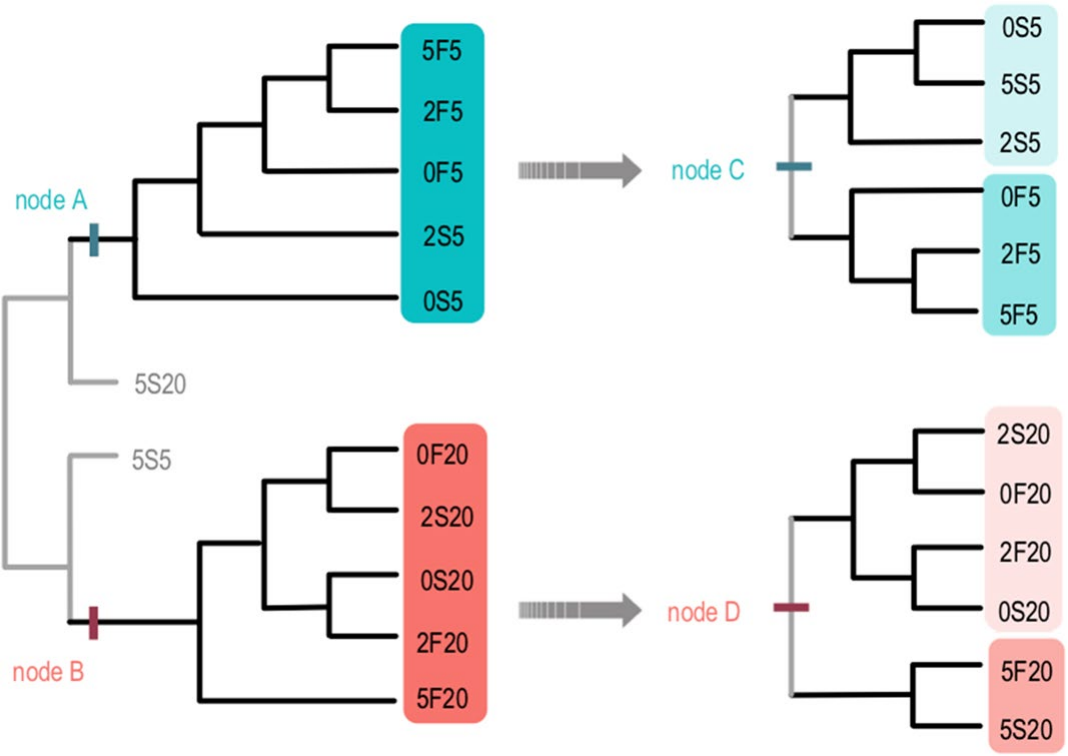
Non-rooted diagrams resulting of the analysis of 93 characters in 12 study groups. Nodes A and B: clusters were formed according to the oxygen tension (20% O_2_ opposing to 5% O_2_); Node C: in 5% O_2_, clusters were formed mainly according to the kinetics of development (Fast vs. Slow) and are shown in shades of blue; Node D: in 20% O_2_, clusters were formed according to the glucose supplementation (0.6 and 2 mM vs. 5 mM) and are represented in shades of coral.

The data matrix analysis of the 93 characters from 12 study groups resulted in a single, fully resolved tree (Fig. 2). The study groups clustered mainly according to the oxygen tension in which the embryos were cultivated (20% O_2_ [Node A] opposing to 5% O_2_ [Node B]), suggesting this category superimposes the others (glucose concentration and kinetics of development). Groups 5S20 and 5S5 present particular characteristics that prevented them from being included in any of the nodes.

In a second step, we isolated the oxygen tension factor and reanalyzed the matrix. The two resulting dendrograms were fully resolved (Fig. 2). Strikingly, in 20% O_2_ (Node D), groups cultured in lower glucose concentration (0.6 mM and 2 mM) clustered opposing to those cultured in a high glucose environment (5 mM). On the other hand, in 5% O_2_ (Node C), the kinetics of development was more decisive. As a consequence, the Fast groups clustered in opposition to the Slow groups regardless of the glucose supplementation.

Our next effort was to identify which attributes were responsible for the clusterization in each of the situations and to understand their roles in the study groups. Thus, the attributes contained in each node and their respective behavior in the main clusters are discussed below.

### Oxygen tension dictates metabolic response during in vitro culture

**Figure 3:**
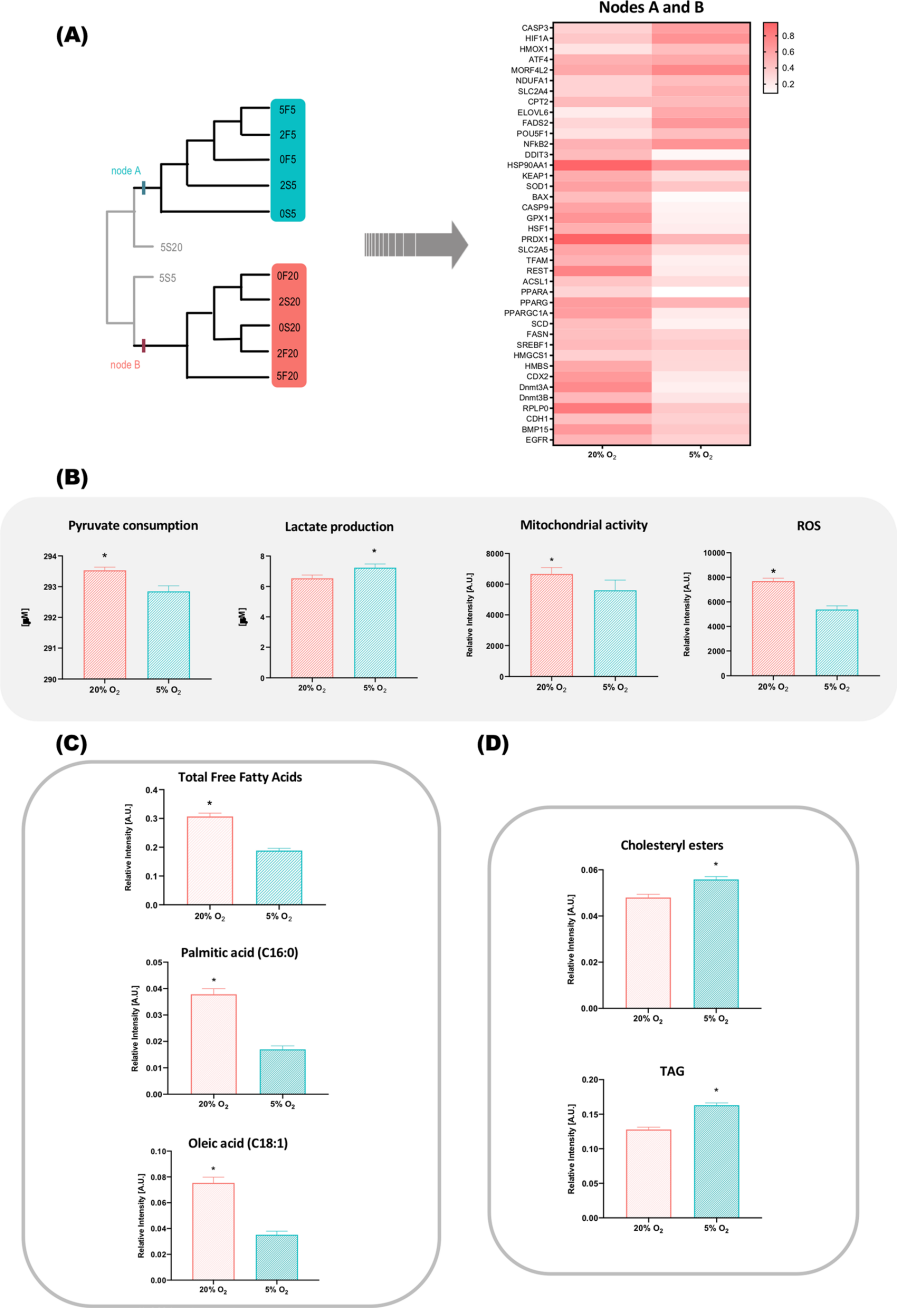
Analysis of the groups cultured at 20% and 5% O_2_. **(A)** Non-rooted diagram generated by cluster analysis and heatmap based on the characters responsible for the formation of nodes A and B. **(B)** Panel showing biochemical evidences evaluated for both 20% and 5% O_2_ groups. **(C)**, **(D)** show the relative amounts of lipids verified for all groups cultured in both oxygen tensions. *Represents p < 0.05.

Oxygen tension plays a central role during the in vitro culture of bovine embryos. The formation of Nodes A and B (Fig. 3A) shows that groups cultured in higher oxygen tension are clustered together mainly due to their active mechanisms of stress response, which is highlighted by the presence of genes related to oxidative (SOD1, GPX1 and PRDX1) and endoplasmic reticulum stress (HSF1 and HSP90), as well as cell death mechanisms (BAX, CASP9 and DDIT3). The stressed metabolism also shows by the presence of reactive oxygen species that are known for directly damaging important transcription regulators such as KEAP1, TFAM, REST, CDX2, Dnmt3a and Dnmt3b. Particularly regarding the amount of ROS, results confirm all groups cultured in 20% O_2_ presented increased amounts of free radicals (Fig. 3B).

Regarding the metabolism of carbohydrates, we verified that the groups cultured in atmospheric oxygen tension had more intense mitochondrial activity and also consumed more pyruvate, which is usually converted to acetyl-CoA in mitochondria and feeds into the TCA cycle. On the other hand, groups cultured in a low oxygen environment increased the conversion of pyruvate to lactate as demonstrated on Fig. 3B.

Another important aspect connecting the groups cultured in a higher oxygen concentration is the lipid metabolism, more specifically the implication of genes involved in the synthesis of fatty acids such as FASN, SCD, PPARG, PPARGC1A, SREBF1 and HMGCS1. This result corroborates the higher relative amounts of total FFA, including palmitic and oleic acids that were found in the groups cultured at 20% O_2_ (Fig. 3C).

On the contrary, genes related to elongation and unsaturation of the fatty acids chain (ELOVL6 and FADS2) were represented in the groups cultured at a lower oxygen environment, which is also in accordance with the higher relative amounts of cholesteryl esters and TAGs found in those groups (Fig. 3D).

### The metabolism of Fast embryos is favored in a hypoxic environment

**Figure 4:**
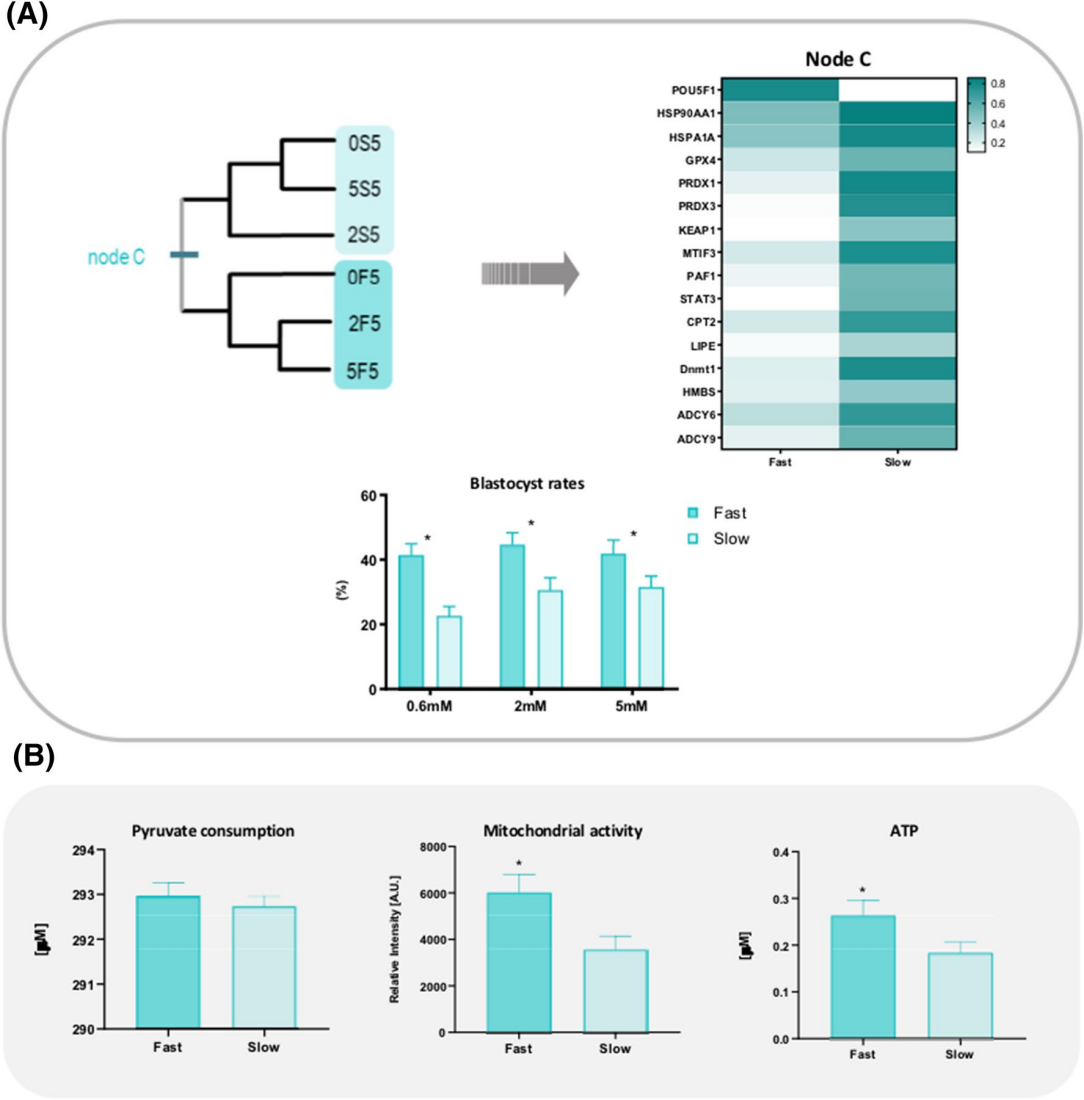
Analysis of Fast and Slow groups cultured at 5% O_2_. **(A)** Non-rooted diagram generated by cluster analysis and heatmap based on the characters responsible for the formation of node D. **(B)** Panel shows the biochemical evidences assessed for both Fast and Slow groups cultured at 5% O_2_. *Represents p < 0.05.

When only the groups cultured at 5% O_2_ were submitted to cluster analysis, we observed an evident separation according to the kinetics of development. Out of the 16 attributes responsible for this clustering (Node D, Fig. 4A), 15 are more represented on Slow groups. Of those, 7 genes are involved with the stress response (HSP90, HSPA1A, PRDX1, PRDX3, GPX4, KEAP1 and STAT3), 2 are involved with DNA methylation mechanisms (Dnmt1 and PAF1), 2 with lipid metabolism (LIPE and CPT2) and 3 to unrelated functions (ADCY6, ADCY9 and MTIF3).

Apart from the differences highlighted in the gene expression pattern, we also observed significantly higher mitochondrial activity for Fast embryos combined with an increased ATP content (Fig. 4B), which probably explains the higher blastocyst rates compared to Slow embryos in all groups, regardless of the glucose concentration in the culture media (Fig. 4B).

### IVP at high oxygen tension: the glucose concentration matters

The groups cultured at a higher oxygen tension were isolated and submitted to cluster analysis, revealing a separation of the groups cultured at 5 mM of glucose (high glucose environment) from the groups cultured in low or physiological levels (0.6 mM and 2 mM).

**Figure 5:**
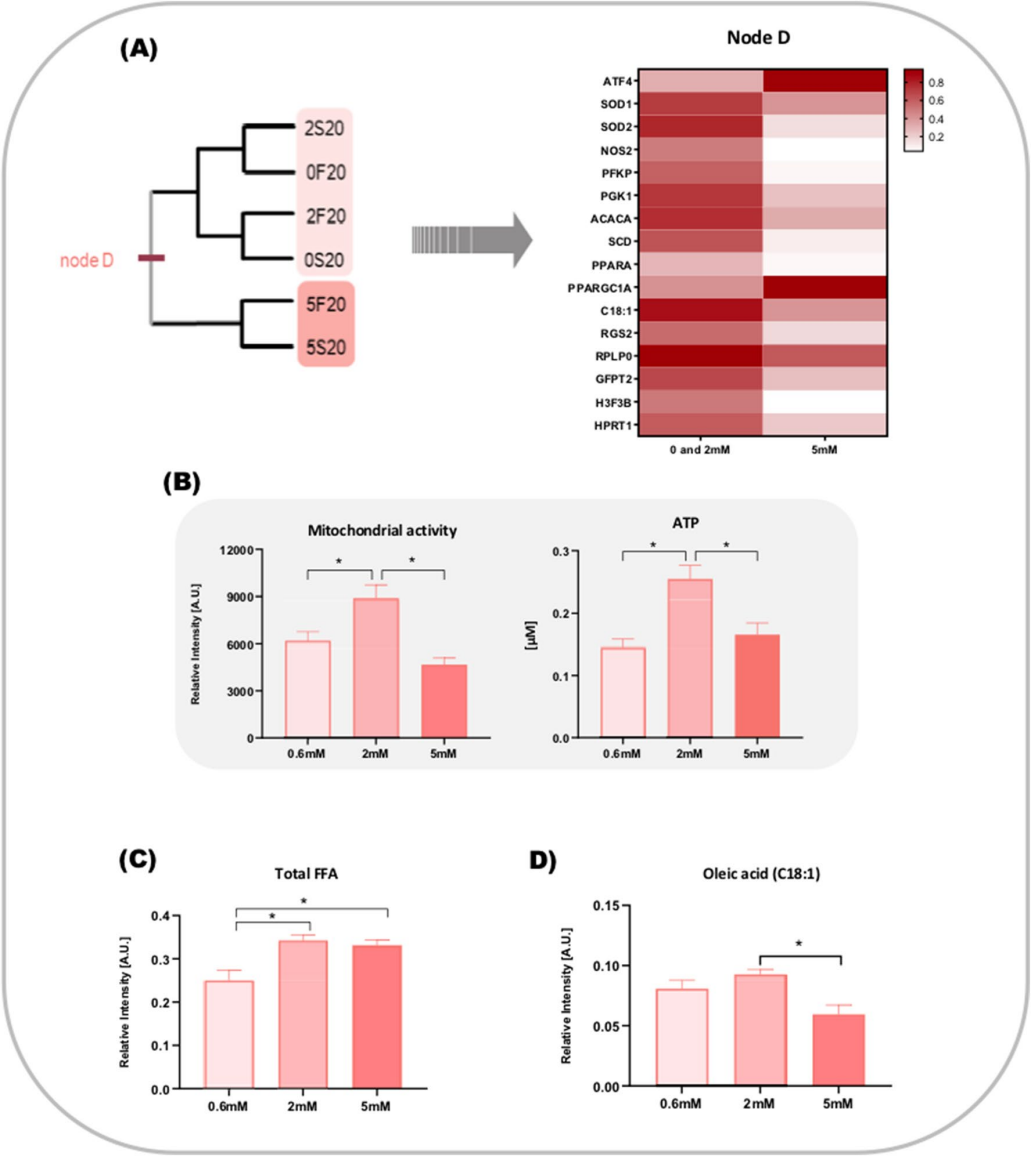
Analysis of the groups cultured in different glucose concentrations at 20% O_2_. **(A)** Non-rooted diagram generated by cluster analysis and heatmap based on the characters responsible for the formation of node C. **(B)** Panel showing biochemical evidences evaluated for groups cultured in different glucose concentrations at 20% O_2_ (C) and (D) show relative amounts of lipids verified for this same groups. *Represents p < 0.05.

In terms of gene expression, the cluster analysis indicates a more active transcription in the groups cultured with lower glucose concentrations. In this case, the node formation (Node C, Fig. 5A) points out to the attributes related to energy production through glycolysis (PFPK and PGK1), as well as to augmented mitochondrial activity in the groups cultured at lower glucose concentrations (0.6 and 2 mM). However, a closer observation of those groups revealed this is a limited effect: mitochondrial activity and ATP content were improved only in embryos cultured at 2 mM (Fig. 5B). Increasing the provision of substrates does not necessarily imply in increasing energy production when embryos are on high oxygen tension.

As previously mentioned, lipid metabolism is a prominent characteristic of the groups cultured in a higher oxygen concentration and a few attributes in node D (ACACA, PPARA, SCD and C18:0) were pointing to this direction. A more detailed analysis of fatty acids behavior showed that relative amount of total FFA was increased in groups culture in 2 and 5 mM of glucose (Fig. 5C). However, specifically for oleic acid we observed a decrease in the groups cultured in high glucose environment (Fig. 5D).

**Figure 6:**
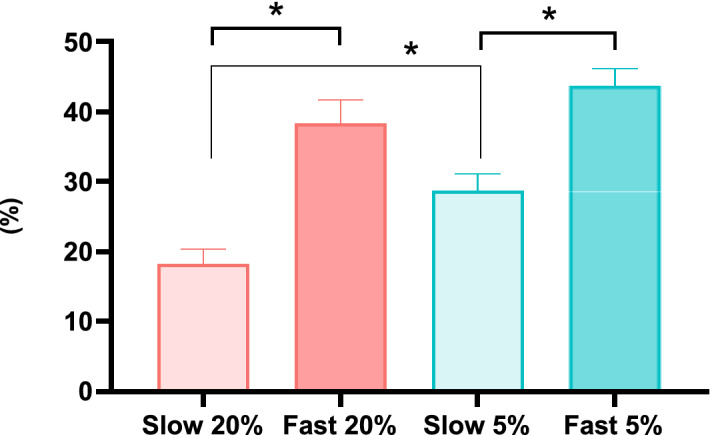
Blastocyst rates calculated for both Fast and Slow groups cultured under high or low oxygen tension. *Represents p < 0.05.

Even if the kinetics of development was not decisive in clustering the groups cultured at 20% O_2_, it clearly affects blastocyst development as the rates in Slow groups were half of the rates in Fast groups. The same was observed for Fast and Slow groups cultured at 5% O_2_. Still, at 5% O_2_, blastocyst rates for Slow groups were around 10% higher than at 20% O_2_, as demonstrated on (Fig. 6).

### The next step: which culture system produces embryos with better energy efficiency?

**Figure 7:**
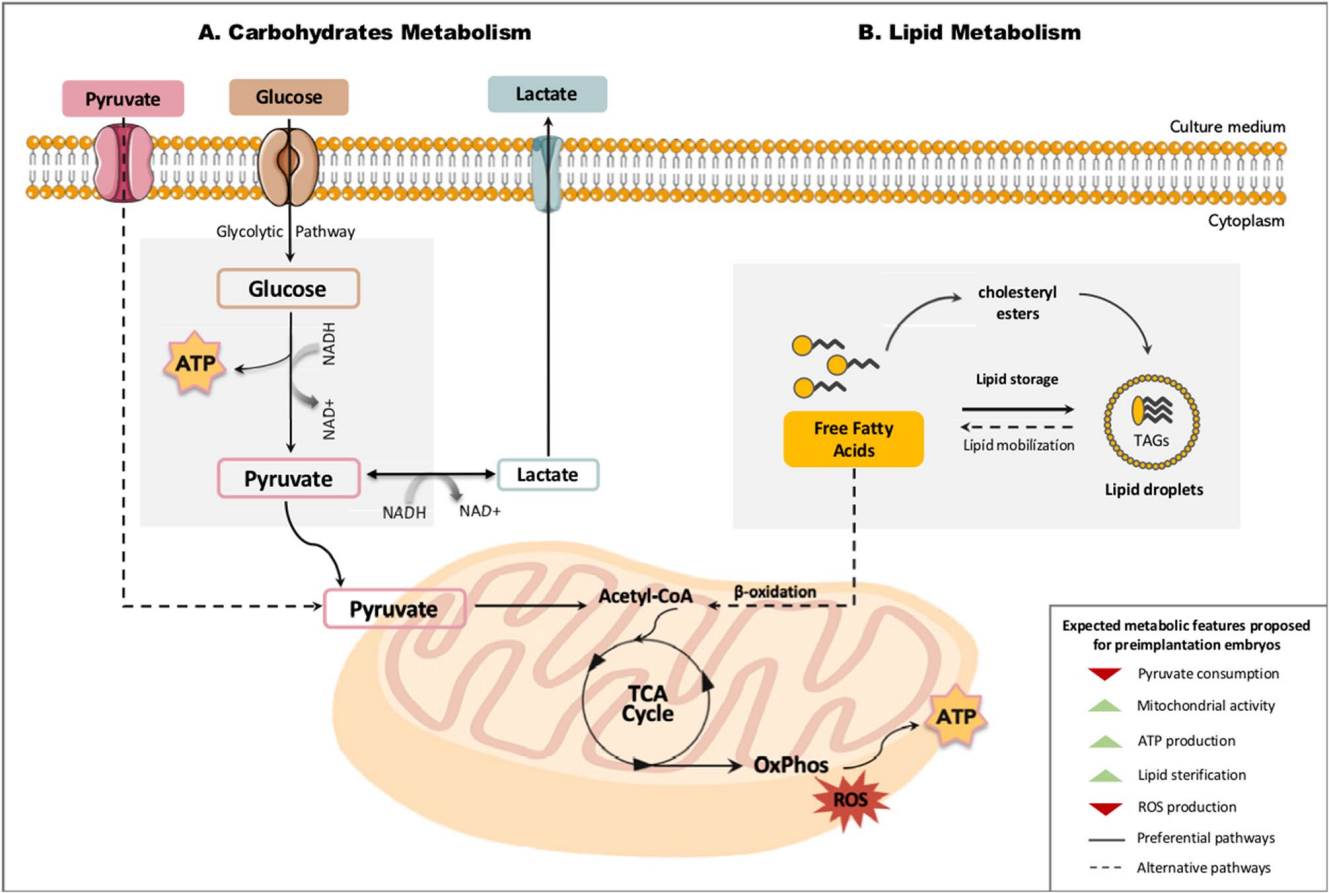
Simplified schematic model proposed for the metabolism of blastocysts cultured in optimized in vitro conditions. The best culture system allows the embryos to obtain pyruvate from glycolysis. As a consequence, there is lower need to consume pyruvate directly from the culture media. Pyruvate is irreversibly converted to acetyl-CoA, which enters the TCA cycle to provide electrons for oxidative phosphorylation and produce ATP. In this context, high ATP production, with low amount of ROS would represent the healthy activity of mitochondria. Finally, in the proposed model, lipid metabolism is driven towards esterification, generating important precursor for membrane synthesis and energy storage.

Exclusively using biochemical evidences measured in this study, we developed a qualitative approach to better visualize the characteristics of the embryos produced in each culture condition. In terms of metabolic competence, the best culture system would be the one that produces embryos with the following characteristics: lower pyruvate consumption, middling to high mitochondrial activity (low production of ROS and high ATP content), and lipid metabolism towards esterification of fatty acids instead of oxidation, as we propose on the schematics of Fig. 7.

It is important to keep in mind that too high or too low levels of mitochondrial membrane potential may induce unwanted loss of cell viability^28^. Therefore, both extremes received a low score regarding mitochondrial activity. Besides, blastocyst rates were included as a marker for the efficiency of each culture system.

**Figure 8:**
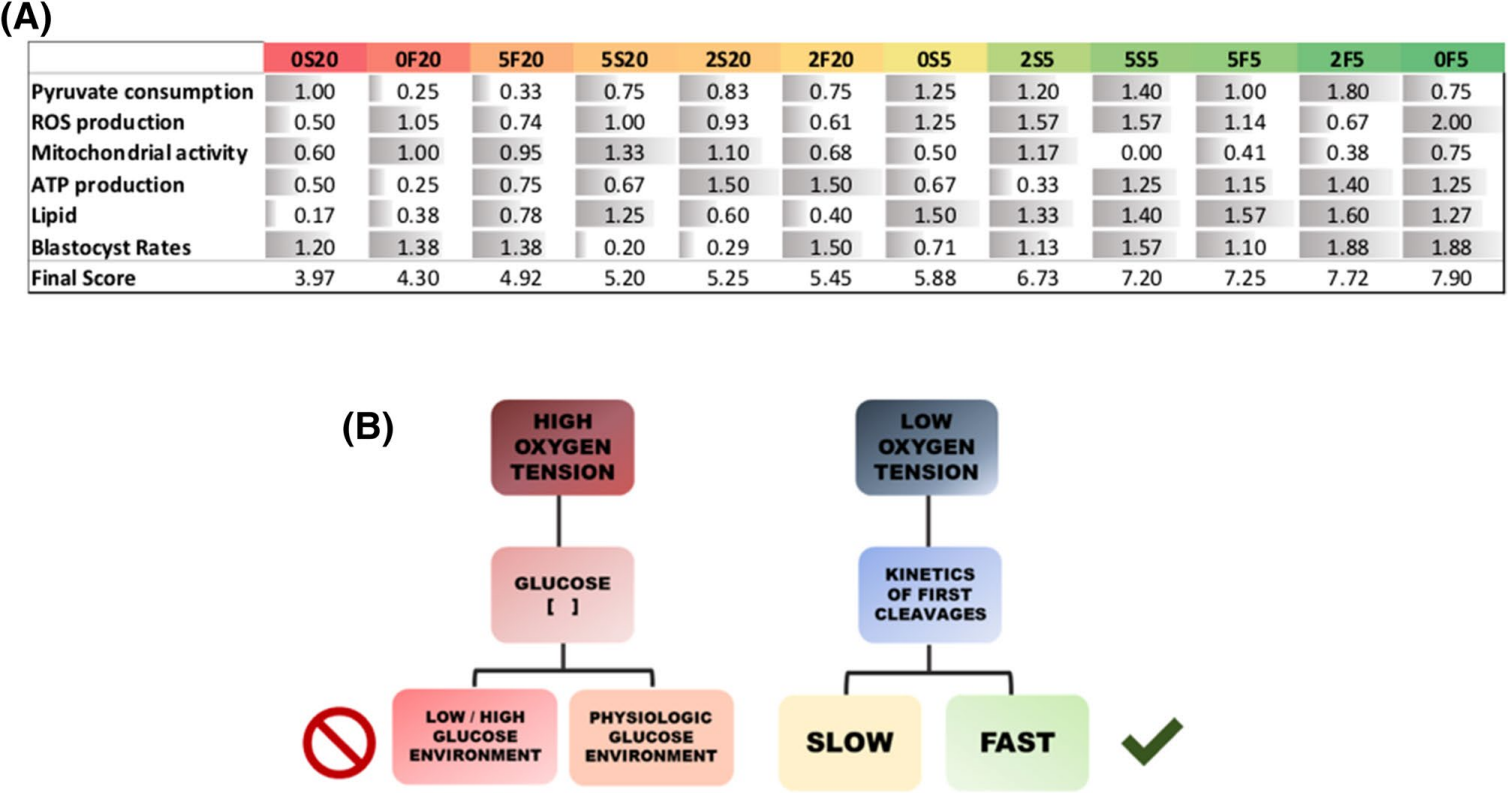
**(A)** Score chart showing the normalized scores for each attribute and the final score (sum of normalized scores) for each study group. **(B)** Hierarchical chart summarizes the ability of culture systems to produce energetically efficient embryos. Fast embryos cultured at low oxygen tension and low glucose concentration showed more of the expected characteristics of such metabolism.

Remarkably, when the scores were assigned, groups clustered in a very similar way as previously happened for hierarchical analysis (Fig. 8A). Groups cultured in higher oxygen tension presented lower final grades, with the lowest values for the embryos cultured in a low glucose environment (0S20 and 0F20). On the contrary, the groups cultured in a hypoxic environment scored better. For these groups, the kinetics of development seems to play an important role, as there is also a clear distinction between Fast and Slow, with the Slow groups presenting the lower scores.

Confirming the results obtained by cluster analysis, the amount of glucose certainly influences the energy efficiency of the embryos. At 20% O_2_, this relationship becomes more evident. Offering a low glucose concentration (~ 0.6 mM in this study) in a high oxygen environment may generate fair blastocyst rates, but not without a compromised metabolic efficiency.

On the other side, the lower oxygen tension seems to foster the metabolism of fast-developing embryos. In terms of lipid metabolism, these embryos prefer the esterification of fatty acids, which can either be stored inside lipid droplets in the form of cholesterol esters and TAG or enter the phospholipids pathway to help build membrane for cell proliferation. Moreover, whilst there is no difference in blastocyst rates among Fast groups cultured at 5% O_2_, there is still some heterogeneity in embryonic response.

Finally, Fast embryos cultured with lower glucose (0.6 mM) in a hypoxic environment were the ones responding more accordingly to a presumable efficient metabolism. These embryos are able to produce a high ATP supply with decreased ROS quantities, thereby indicating functional mitochondria. These results were summarized in Fig. 8B, indicating the ability of each culture system to produce energetically efficient embryos.

## Discussion

Although other studies have tackled issues such as how culture under low oxygen produces more efficient embryos and why Fast embryos perform better, the broad approach we address here is unique. Previous reports stated numerous differences in the transcription pattern and DNA methylation profile between bovine embryos developing at different cleavage speeds^15,18^. Other studies provided evidences that such differences were related to the metabolic stress caused by the culture system^16,17^. Based on these assumptions, the present study was initially designed to investigate how Fast and Slow embryos respond to stressors commonly found during in vitro culture, i.e., non-optimized oxygen tension and high glucose concentration.

The concentration of glucose in the bovine oviduct fluid (~ 2 mM) is lower than in blood plasma (~ 5.5 mM), indicating a differential secretion of this substrate for adaptation to embryonic metabolism^29^. Similarly, the lumen of the female reproductive tract provides an oxygen concentration of 2–8%, differing from the atmospheric level of 20%^6^. The oviduct also provides an efficient oviduct red/ox system, which guarantees an ideal environment for the passage of the embryo^30^.

The relevance of energy metabolism to control gene expression, since cells constantly adjust their metabolic state in response to extracellular signaling and nutrient availability, was previously demonstrated^31^. Therefore, by combining environmental stressors and embryos with different phenotypes, we were able to better visualize this plasticity during initial development. When all the study groups were analyzed together, cluster analysis revealed an evident metabolic signature, showing the modulation of embryonic metabolism is highly related to the oxygen tension in the culture system.

Previous studies performed with in vitro produced embryos from different species, including cattle, indicated that lower oxygen tension results in better embryonic development, with increased cleavage and blastocyst rates, and higher post-implantation success rate^23,32–34^. According to these same reports, the exposure to different oxygen concentration generates embryos with distinct transcription profiles. For the bovine embryos herein examined, we observed that not only the profile of gene expression differs, but a greater number of genes are responsible for such differences on groups cultured in high oxygen. This may be related to the activation of adaptive responses, as described by Leese^2^ for human embryos. Thus, while mammalian embryos are quite susceptible to the effects of high oxygen concentration, having an active metabolism could help them compensate for damages caused by environmental injuries and restore developmental trajectory.

Furthermore, the genes represented in nodes A and B were mostly related to oxidative and reticulum stress response pathways such as those mediated by GPX1, PRDX1^35,36^ and Heat Shock Proteins (HSF1 and HSP90)^37^. The exposure to high levels of oxygen is a known source of oxidative stress^33^ and has also been associated to the activation of pro-apoptotic signaling cascades (CASP9, BAX and DDIT3)^34^.

Supporting these findings, an expected higher presence of ROS was confirmed in the groups cultured at 20% O_2_. Among possible damages caused by the excess of ROS, we highlight changes in the expression of transcription factors, such as REST, PAF1, STAT3, CDX2, KEAP1 and TFAM. In general, mRNAs and other nucleic acids are easily targeted by free radicals, which leads to detrimental effects in cellular machinery and protein synthesis^2^. These results also reinforce recently published data describing the differences in the control of gene transcription mediated by the oxygen tension^23^.

Along with the metabolism of the embryos cultured in high oxygen, our analysis clearly indicated the importance of energy substrates availability. The results show a higher consumption of pyruvate at 20% O_2_, which corroborates the representation of glycolysis-related genes (PFKP and PGK1) in those groups. However, this effect was clearly not widespread. Only the groups cultured in physiological glucose concentration (2 mM) presented improvements in mitochondrial activity and ATP amounts, suggesting there is an optimal amount of energy substrates that must be available to the embryos. This is in agreement with Leese et al.^4^, who proposed that embryos on an intermediary range of pyruvate consumption are more likely to form blastocysts.

When oxygen is sparse, cells adapt to hypoxia by reprogramming the expression of a number of genes involved in carbohydrate metabolism^38,39^. Hypoxia also enhances anaerobic energy production. To reduce mitochondrial function, and therefore decrease oxygen consumption and ROS production, hypoxia induced factors block the conversion of pyruvate. Then, the flow of pyruvate into the mitochondria decreases, fueling the production of lactate and reducing mitochondrial metabolic processes^40^. Such events are evident in the embryos cultured at low oxygen that presented lower pyruvate consumption coupled to an increased lactate production.

Groups cultured in a high oxygen tension also presented differential lipid metabolism. A number of genes responsible for the synthesis of fatty acids were found to be important in those groups. Accordingly, we verified increased relative amounts of total free fatty acids, and particularly of stearic and oleic acids when compared to groups cultured in 5% O_2_. The excess of intracellularly accumulated free fatty acids can cause lipotoxicity^40^. To avoid this, cells can convert FFA into neutral TAGs that are stored in lipid droplets and serve as energy depots^41^. Indeed, the activation of such mechanism was observed in our embryos cultured in low oxygen and it is also typical in tumoral cells. It prevents the formation of free radicals, with the lipid droplets serving as building blocks for the production of essential sterol esters and phospholipids required in high proliferative cells for the biogenesis of new membranes^35^.

Results indicate that the metabolism of Fast embryos is favored in a 5% O_2_ environment, which is depicted by the higher mitochondrial activity and ATP content when compared to Slow embryos. This suggests a very thin threshold separating two types of embryos with high metabolic demands: (i) embryos with higher activity due to loss of transcriptional control; and (ii) embryos with a certain degree of damage but still able to activate adaptive pathways, surviving even with poor quality, which may lead to future implications.

We believe Slow embryos belong to the first type. Despite having a more active metabolism, the conversion rates to blastocyst were lower in all Slow embryos when compared to the faster counterparts. Considering that the DNA from Slow blastocysts is significantly less methylated than DNA from Fast blastocyst^18^, we speculate that the loss of transcriptional control and the lower ability to activate adaptive pathways may be explained by perturbations in epigenetic mechanisms.

Finally, the score system herein created reflects how the external environment impacts and helps defining the energy efficiency of the embryos. Moreover, it allows us to propose the conditions that better support the development of these embryos in vitro. In accordance with the Dynamic Energy Budget theory, our scores consider “efficiency” in the energy sense, meaning that for any given substrate, there is an optimal range of metabolic activity to support embryonic demand, but when exposed to a challenge or stress, the resulting response will have an energy cost that will make them shift out of this metabolic range^42^. Therefore, we propose that more viable embryos are those capable of producing more energy with minimum disturbances to the energy production system as whole, i.e. the mitochondria and lipid metabolism.

Although the high oxygen tension still allows the formation of blastocysts, the energy production of these groups is clearly distressed; hence, they presented the lowest score values. In other words, the metabolism is able to rectify the stress enough to maintain these embryos alive (somatic maintenance), however, it may lead substrates away from other essential processes or even boost additional activity to increase embryonic complexity (i.e. differentiation), as suggested by previous publications^42,43^. If further resources are required, development might be compromised as indicated by their metabolic phenotype.

The highest scores belong to the groups cultured at 5% O_2_. Under an oxygen condition apparently favoring energy metabolism, the heterogeneous response between Fast and Slow embryos becomes more obvious. The lowest scores belong to Slow embryos suggesting their deficient energy metabolism is consequence of other phenotype-related mechanisms and not driven by external environment. Among the Fast groups, the one more closely matching the expected characteristics of an efficient metabolism is the group 0F5. The combination of lower glucose and oxygen tension offered to this group are probably just the right amount to boost their energy production and sustain the demands of a healthier developmental program.

It is relevant to consider that these characteristics are representative of the physiology, but do not represent the potential of these embryos to establish pregnancy in the event of a transfer to recipient cows. It is possible that embryos produced in vitro could show some compensation after entering the uterine environment that would allow them to implant and develop into a calf. The metabolic imbalance suffered in vitro could be overcome, but likely not without consequences for the future offspring.

In conclusion, our analyses depict how embryonic metabolic response can be quite heterogeneous depending on early phenotypes and composition of the culture media surrounding the embryos. This is a pioneer study that simultaneously shows the capacity embryos have to switch to other substrates when needed, and to modulate molecular machinery, ensuring their survival even in the face of adverse and non-optimized external conditions. We believe that the information presented here may greatly contribute to decision making in terms of choosing the more suitable culture system or performing interventions that favor specific characteristics and rescue embryos that would otherwise perish. Future studies must focus on determining whether these embryos have the same implantation potential and what are the consequences of an inefficient energy metabolism for the blastocyst in a short term, and also for the future offspring in a long term.

## Supplementary information

Supplementary Information.

## References

[1] Leese HJ, Sturmey RG, Baumann CG, McEvoy TG (2007). Embryo viability and metabolism: Obeying the quiet rules. Hum. Reprod..

[2] Leese HJ, Baumann CG, Brison DR, McEvoy TG, Sturmey RG (2008). Metabolism of the viable mammalian embryo: Quietness revisited. Mol. Hum. Reprod..

[3] Gardner DK, Wale PL, Collins R, Lane M (2011). Glucose consumption of single post-compaction human embryos is predictive of embryo sex and live birth outcome. Hum. Reprod..

[4] Leese HJ (2016). Biological optimization, the Goldilocks principle, and how much is lagom in the preimplantation embryo. Mol. Reprod. Dev..

[5] Leese HJ (2012). Metabolism of the preimplantation embryo: 40 years on. Reproduction.

[6] Gardner DK, Harvey AJ (2015). Blastocyst metabolism. Reprod. Fertil. Dev..

[7] Rollo C, Li Y, Jin XL, O’Neill C (2017). Histone 3 lysine 9 acetylation is a biomarker of the effects of culture on zygotes. Reproduction.

[8] Vigneault C, Gravel C, Vallée M, McGraw S, Sirard M-A (2009). Unveiling the bovine embryo transcriptome during the maternal-to-embryonic transition. Reproduction.

[9] Sutton-McDowall ML, Feil D, Robker RL, Thompson JG, Dunning KR (2012). Utilization of endogenous fatty acid stores for energy production in bovine preimplantation embryos. Theriogenology.

[10] Krisher RL, Prather RS (2012). A role for the Warburg effect in preimplantation embryo development: Metabolic modification to support rapid cell proliferation. Mol. Reprod. Dev..

[11] Cagnone G, Sirard M-A (2016). The embryonic stress response to in vitro culture: Insight from genomic analysis. Reproduction.

[12] Silva-Rodriguez, J., Colomer, A., Meseguer, M. & Naranjo, V. Predicting the success of blastocyst implantation from morphokinetic parameters estimated through CNNs and sum of absolute differences. in *2019 27th European Signal Processing Conference (EUSIPCO)* 1–5 (IEEE, 2019). 10.23919/EUSIPCO.2019.8902520.

[13] Cruz M (2012). Timing of cell division in human cleavage-stage embryos is linked with blastocyst formation and quality. Reprod. Biomed. Online.

[14] Zaninovic N (2019). A comparison of morphokinetic markers predicting blastocyst formation and implantation potential from two large clinical data sets. J. Assist. Reprod. Genet..

[15] Milazzotto MP (2016). Early cleavages influence the molecular and the metabolic pattern of individually cultured bovine blastocysts. Mol. Reprod. Dev..

[16] Silva T (2016). Morphokinetic-related response to stress in individually cultured bovine embryos. Theriogenology.

[17] dos Santos ÉC, de Lima CB, Annes K, Milazzotto MP (2016). Noninvasive characterization of metabolites secreted in culture media by bovine embryos during in vitro production. Metabolomics.

[18] Ispada J (2018). Genome-wide screening of DNA methylation in bovine blastocysts with different kinetics of development. Epigenet. Chromatin.

[19] Parrish JJ, Susko-Parrish J, Winer MA, First NL (1988). Capacitation of bovine sperm by heparin. Biol. Reprod..

[20] Vajta G (2000). New method for culture of zona-included or zona-free embryos: The well of the well (WOW) system. Mol. Reprod. Dev..

[21] Annes, K., Soares, C. A., Lima, C. de & Milazzotto, M. P. Effective individual culture system for in vitro production of bovine embryos (2017). 10.11606/issn.1678-4456.bjvras.2017.107721.

[22] de Lima CB (2018). Comprehensive lipid profiling of early stage oocytes and embryos by MRM profiling. J. Mass Spectrom..

[23] Leite, R. F. *et al.* Oxidative stress alters the profile of transcription factors related to early development on in vitro produced embryos. In *Oxidative Medicine and Cellular Longevity,* https://www.hindawi.com/journals/omcl/2017/1502489/ (2017) 10.1155/2017/1502489. 10.1155/2017/1502489 PMC5676474 29209446

[24] Andersen CL, Jensen JL, Ørntoft TF (2004). Normalization of real-time quantitative reverse transcription-PCR data: A model-based variance estimation approach to identify genes suited for normalization, applied to bladder and colon cancer data sets. Cancer Res..

[25] Kitching, I. J., Forey, P., Humphries, C. & Williams, D. *Cladistics: The Theory and Practice of Parsimony Analysis*. (Oxford University Press, Oxford, 1998).

[26] Goloboff PA, Farris JS, Nixon KC (2008). TNT, a free program for phylogenetic analysis. Cladistics.

[27] Letunic I, Bork P (2016). Interactive tree of life (iTOL) v3: An online tool for the display and annotation of phylogenetic and other trees. Nucleic Acids Res..

[28] Zorova LD (2018). Mitochondrial membrane potential. Anal. Biochem..

[29] Hugentobler SA, Humpherson PG, Leese HJ, Sreenan JM, Morris DG (2008). Energy substrates in bovine oviduct and uterine fluid and blood plasma during the oestrous cycle. Mol. Reprod. Dev..

[30] Guérin P, El Mouatassim S, Ménézo Y (2001). Oxidative stress and protection against reactive oxygen species in the pre-implantation embryo and its surroundings. Hum. Reprod. Update.

[31] Vander Heiden, M. G., Cantley, L. C. & Thompson, C. B. Understanding the Warburg effect: The metabolic requirements of cell proliferation. *Science* **324**, 1029–1033 (2009). 10.1126/science.1160809 PMC2849637 19460998

[32] Amin A (2014). Bovine embryo survival under oxidative-stress conditions is associated with activity of the NRF2-mediated oxidative-stress-response pathway. Mol. Reprod. Dev..

[33] Gad A (2012). Molecular mechanisms and pathways involved in bovine embryonic genome activation and their regulation by alternative in vivo and in vitro culture conditions. Biol. Reprod..

[34] Yoon S-B (2014). Developmental competence of bovine early embryos depends on the coupled response between oxidative and endoplasmic reticulum stress. Biol. Reprod..

[35] Ufer, C. & Wang, C. C. The roles of glutathione peroxidases during embryo development. *Front. Mol. Neurosci.* **4** (2011). 10.3389/fnmol.2011.00012 PMC3148772 21847368

[36] Leyens G, Knoops B, Donnay I (2004). Expression of peroxiredoxins in bovine oocytes and embryos produced in vitro. Mol. Reprod. Dev..

[37] Balasubramanian S (2007). Expression pattern of oxygen and stress-responsive gene transcripts at various developmental stages of in vitro and in vivo preimplantation bovine embryos. Theriogenology.

[38] Samanta D, Semenza GL (2018). Metabolic adaptation of cancer and immune cells mediated by hypoxia-inducible factors. Biochim. Biophys. Acta Rev. Cancer.

[39] Xie H, Simon MC (2017). Oxygen availability and metabolic reprogramming in cancer. J. Biol. Chem..

[40] Mylonis, I., Simos, G. & Paraskeva, E. Hypoxia-inducible factors and the regulation of lipid metabolism. *Cells* **8** (2019). 10.3390/cells8030214 PMC6468845 30832409

[41] Wang H, Airola MV, Reue K (2017). How lipid droplets ‘TAG’ along: Glycerolipid synthetic enzymes and lipid storage. Biochim. Biophys. Acta Mol. Cell Biol. Lipids.

[42] Guerif F, McKeegan P, Leese HJ, Sturmey RG (2013). A simple approach for COnsumption and RElease (CORE) analysis of metabolic activity in single mammalian embryos. PLoS ONE.

[43] Sousa, T., Domingos, T. & Kooijman, S. A. L. M. From empirical patterns to theory: A formal metabolic theory of life. *Philos. Trans. R. Soc. B: Biol. Sci.* **363**, 2453–2464 (2008). 10.1098/rstb.2007.2230 PMC2606805 18331988

