# Supplementary material for: The dynamics between in vitro culture and metabolism: embryonic adaptation to environmental changes
Source: Sci Rep. 2020 Sep 24;10:15672. doi: 10.1038/s41598-020-72221-1 (PMC7518437; doi:10.1038/s41598-020-72221-1)
Supplement: Supplementary file 1 — Supplementary Information. [file 41598_2020_72221_MOESM1_ESM.docx]

**Title:** The dynamics between *in vitro* culture and metabolism: embryonic adaptation to environmental changes.

**Authors:** Camila Bruna de Lima^1.2^. Érika Cristina dos Santos^2^. Jéssica Ispada^1.2^. Patrícia Kubo Fontes^3^ Marcelo Fábio Gouveia Nogueira^3.4^. Charles Morphy Dias dos Santos^2^. Marcella Pecora Milazzotto*^1.2^

**Department affiliations:**

1 – Institute of Biomedical Sciences/ University of São Paulo

2 – Center for Natural and Human Sciences / Federal University of ABC

3 – Laboratory FitoFarmaTec. Department of Pharmacology. Institute of Biosciences / São Paulo State University. Campus Botucatu

4 – Department of Biological Sciences. School of Sciences and Languages / São Paulo State University. Campus Assis

***Corresponding author**: Marcella Pecora Milazzotto [(mazamila@gmail.com)](mailto:(mazamila@gmail.com))

Federal University of ABC – Center for Natural and Human Sciences.

Av. dos Estados. 5001. Bairro Santa Terezinha. Bloco A. Lab 502-3

Santo André – SP. Brazil

CEP: 09210-580

**Supplementary table 1:** List of the 178 MRMs selected after the screening step of lipid profiling.

| **Compound Name** | **Precursor Ion** | **Product Ion** |
| --- | --- | --- |
| 14:1 Cholesteryl ester | 612.5 | 369.1 |
| 14:0 Cholesteryl ester | 614.6 | 369.1 |
| 15:1 Cholesteryl ester | 626.6 | 369.1 |
| 15:0 Cholesteryl ester | 628.6 | 369.1 |
| 16:3 Cholesteryl ester | 636.5 | 369.1 |
| 16:2 Cholesteryl ester. zymosteryl palmitoleate | 638.6 | 369.1 |
| 16:1 Cholesteryl ester | 640.6 | 369.1 |
| 16:0 Cholesteryl ester | 642.6 | 369.1 |
| 16:3 Campesteryl ester | 650.6 | 369.1 |
| 16:2 Campesteryl ester | 652.6 | 369.1 |
| 16:1 Campesteryl ester | 654.6 | 369.1 |
| 16:0 Campesteryl ester | 656.6 | 369.1 |
| 16:3 Stigmasteryl ester | 662.6 | 369.1 |
| 18:3 Cholesteryl ester. 16:2 Stigmasteryl ester. 16:3 Sitosteryl ester | 664.6 | 369.1 |
| 18:2 Cholesteryl ester. zymosteryl oleate. 16:1 Stigmasteryl ester. 16:2 Sitosteryl ester | 666.6 | 369.1 |
| 18:1 Cholesteryl ester. 16:0 Stigmasteryl ester. 16:1 Sitosteryl ester | 668.6 | 369.1 |
| 18:0 Cholesteryl ester. 16:0 Sitosteryl ester | 670.6 | 369.1 |
| 18:3 Campesteryl ester | 678.6 | 369.1 |
| lanosteryl palmitoleate. 18:2 Campesteryl ester | 680.6 | 369.1 |
| 18:1 Campesteryl ester | 682.6 | 369.1 |
| 18:0 Campesteryl ester | 684.6 | 369.1 |
| 20:5 Cholesteryl ester | 688.6 | 369.1 |
| 20:4 Cholesteryl ester. 18:3 Stigmasteryl ester | 690.6 | 369.1 |
| 20:3 Cholesteryl ester. 18:2 Stigmasteryl ester. 18:3 Sitosteryl ester | 692.6 | 369.1 |
| 20:2 Cholesteryl ester. 18:1 Stigmasteryl ester. 18:2 Sitosteryl ester | 694.6 | 369.1 |
| 20:1 Cholesteryl ester. 18:0 Stigmasteryl ester. 18:1 Sitosteryl ester | 696.6 | 369.1 |
| 20:0 Cholesteryl ester. 18:0 Sitosteryl ester | 698.6 | 369.1 |
| 20:3 Campesteryl ester | 706.6 | 369.1 |
| lanosteryl oleate. 20:2 Campesteryl ester | 708.6 | 369.1 |
| 20:1 Campesteryl ester | 710.6 | 369.1 |
| Cholesteryl nitrolinoleate | 711.6 | 369.1 |
| 20:0 Campesteryl ester | 712.7 | 369.1 |
| 22:6 Cholesteryl ester | 714.6 | 369.1 |
| 22:5 Cholesteryl ester | 716.6 | 369.1 |
| 22:4 Cholesteryl ester. 20:3 Stigmasteryl ester | 718.6 | 369.1 |
| ecdysone palmitate. 22:3 Cholesteryl ester. 20:2 Stigmasteryl ester. 20:3 Sitosteryl ester | 720.5 | 369.1 |
| Cholesteryl 11-hydroperoxy-eicosatetraenoate. 22:2 Cholesteryl ester. 20:1 Stigmasteryl ester. 20:2 Sitosteryl ester | 722.6 | 369.1 |
| 22:1 Cholesteryl ester. 20:0 Stigmasteryl ester. 20:1 Sitosteryl ester | 724.7 | 369.1 |
| 22:0 Cholesteryl ester. 20:0 Sitosteryl ester | 726.7 | 369.1 |
| 22:3 Campesteryl ester | 734.6 | 369.1 |
| 22:2 Campesteryl ester | 736.7 | 369.1 |
| 22:1 Campesteryl ester | 738.7 | 369.1 |
| 22:0 Campesteryl ester | 740.7 | 369.1 |
| 22:3 Stigmasteryl ester | 746.6 | 369.1 |
| 22:2 Stigmasteryl ester. 22:3 Sitosteryl ester | 748.7 | 369.1 |
| 22:1 Stigmasteryl ester. 22:2 Sitosteryl ester | 750.7 | 369.1 |
| 24:1 Cholesteryl ester. 22:0 Stigmasteryl ester. 22:1 Sitosteryl ester | 752.7 | 369.1 |
| 22:0 Sitosteryl ester | 754.7 | 369.1 |
| C12:0 | 199.2 | 199.2 |
| C12:1 | 197.2 | 197.2 |
| C14:0 | 227.3 | 227.3 |
| C14:1 | 225.2 | 225.2 |
| C16:0 | 255.3 | 255.3 |
| C16:1 | 253.3 | 253.3 |
| C18:0 | 283.3 | 283.3 |
| C18:1 | 281.3 | 281.3 |
| C18:2 | 279.3 | 279.3 |
| C18:3 | 277.3 | 277.3 |
| C18:4 | 275.3 | 275.3 |
| C20:0 | 311.3 | 311.3 |
| C20:1 | 309.3 | 309.3 |
| C20:3 | 305.3 | 305.3 |
| C20:4 | 303.3 | 303.3 |
| C20:5 | 301.3 | 301.3 |
| C22:0 | 339.3 | 339.3 |
| C22:1 | 337.3 | 337.3 |
| C22:4 | 331.3 | 331.3 |
| C22:5 | 329.3 | 329.3 |
| C22:6 | 327.3 | 327.3 |
| C24:0 | 367.3 | 367.3 |
| C24:1 | 365.3 | 365.3 |
| C24:5 | 353.3 | 353.3 |
| C24:6 | 355.3 | 355.3 |
| C26:0 | 395.3 | 395.3 |
| C26:1 | 393.3 | 393.3 |
| C28:0 | 423.4 | 423.4 |
| C30:0 | 451.4 | 451.4 |
| C32:0 | 479.4 | 479.4 |
| C34:0 | 507.5 | 507.5 |
| TAG(48:0)_FA16:0 | 824.8 | 551.8 |
| TAG(48:1)_FA16:0 | 822.8 | 549.8 |
| TAG(48:1)_FA16:1 | 822.8 | 551.8 |
| TAG(48:1)_FA18:1 | 822.8 | 523.8 |
| TAG(48:2)_FA16:0 | 820.8 | 547.8 |
| TAG(48:2)_FA16:1 | 820.8 | 549.8 |
| TAG(48:2)_FA18:1 | 820.8 | 521.8 |
| TAG(48:2)_FA18:2 | 820.8 | 523.8 |
| TAG(50:0)_FA16:0 | 852.8 | 579.8 |
| TAG(50:0)_FA18:0 | 852.8 | 551.8 |
| TAG(50:1)_FA16:0 | 850.8 | 577.8 |
| TAG(50:1)_FA16:1 | 850.8 | 579.8 |
| TAG(50:1)_FA18:0 | 850.8 | 551.8 |
| TAG(50:1)_FA18:1 | 850.8 | 553.8 |
| TAG(50:2)_FA16:0 | 850.8 | 577.8 |
| TAG(50:2)_FA16:1 | 850.8 | 579.8 |
| TAG(50:2)_FA18:1 | 850.8 | 551.8 |
| TAG(50:2)_FA18:2 | 850.8 | 553.8 |
| TAG(50:3)_FA16:0 | 848.8 | 575.8 |
| TAG(50:3)_FA16:1 | 848.8 | 577.8 |
| TAG(50:3)_FA18:1 | 848.8 | 549.8 |
| TAG(50:3)_FA18:2 | 848.8 | 551.8 |
| TAG(52:0)_FA16:0 | 880.8 | 607.8 |
| TAG(52:0)_FA18:0 | 880.8 | 579.8 |
| TAG(52:1)_FA16:0 | 878.8 | 605.8 |
| TAG(52:1)_FA18:0 | 878.8 | 577.8 |
| TAG(52:1)_FA18:1 | 878.8 | 579.8 |
| TAG(52:2)_FA16:0 | 876.8 | 603.8 |
| TAG(52:2)_FA16:1 | 876.8 | 605.8 |
| TAG(52:2)_FA18:0 | 876.8 | 575.8 |
| TAG(52:2)_FA18:1 | 876.8 | 577.8 |
| TAG(52:2)_FA18:2 | 876.8 | 579.8 |
| TAG(52:3)_FA16:0 | 874.8 | 601.8 |
| TAG(52:3)_FA16:1 | 874.8 | 603.8 |
| TAG(52:3)_FA18:1 | 874.8 | 575.8 |
| TAG(52:3)_FA18:2 | 874.8 | 577.8 |
| TAG(52:4)_FA16:0 | 872.8 | 599.8 |
| TAG(52:4)_FA18:1 | 872.8 | 573.8 |
| TAG(52:4)_FA18:2 | 872.8 | 575.8 |
| TAG(52:4)_FA20:4 | 872.8 | 551.8 |
| TAG(54:1)_FA16:0 | 906.8 | 633.8 |
| TAG(54:1)_FA18:0 | 906.8 | 605.8 |
| TAG(54:1)_FA18:1 | 906.8 | 607.8 |
| TAG(54:2)_FA16:0 | 904.8 | 631.8 |
| TAG(54:2)_FA18:0 | 904.8 | 603.8 |
| TAG(54:2)_FA18:1 | 904.8 | 605.8 |
| TAG(54:2)_FA18:2 | 904.8 | 607.8 |
| TAG(54:3)_FA16:0 | 902.8 | 629.8 |
| TAG(54:3)_FA18:0 | 902.8 | 601.8 |
| TAG(54:3)_FA18:1 | 902.8 | 603.8 |
| TAG(54:3)_FA18:2 | 902.8 | 605.8 |
| TAG(54:4)_FA16:0 | 900.8 | 627.8 |
| TAG(54:4)_FA18:1 | 900.8 | 601.8 |
| TAG(54:4)_FA18:2 | 900.8 | 603.8 |
| TAG(54:4)_FA20:4 | 900.8 | 579.8 |
| TAG(54:5)_FA16:0 | 898.8 | 625.8 |
| TAG(54:5)_FA18:1 | 898.8 | 599.8 |
| TAG(54:5)_FA18:2 | 898.8 | 601.8 |
| TAG(54:5)_FA20:4 | 898.8 | 577.8 |
| TAG(54:8)_FA18:1 | 892.8 | 593.8 |
| TAG(56:3)_FA18:1 | 930.8 | 631.8 |
| TAG(56:3)_FA18:2 | 930.8 | 633.8 |
| TAG(56:4)_FA16:0 | 928.8 | 655.8 |
| TAG(56:5)_FA16:0 | 926.8 | 653.8 |
| TAG(56:5)_FA18:1 | 926.8 | 627.8 |
| TAG(56:5)_FA18:2 | 926.8 | 629.8 |
| TAG(56:6)_FA18:1 | 924.8 | 625.8 |
| TAG(56:6)_FA20:4 | 924.8 | 603.8 |
| TAG(56:8)_FA18:1 | 920.8 | 621.8 |
| TAG(58:1)_FA18:1 | 962.9 | 663.9 |
| TAG(58:2)_FA18:1 | 960.9 | 661.9 |
| TAG(58:2)_FA18:2 | 960.9 | 663.9 |
| TAG(58:5)_FA18:1 | 954.8 | 655.8 |
| TAG(58:6)_FA18:1 | 952.8 | 653.8 |
| TAG(58:7)_FA20:0 | 950.8 | 621.8 |
| TAG(60:0)_FA20:0 | 992.9 | 663.9 |
| TAG(60:4)_FA20:4 | 984.9 | 663.9 |
| TAG(48:0)_FA16:0 | 824.8 | 551.8 |
| TAG(48:1)_FA16:0 | 822.8 | 549.8 |
| TAG(48:1)_FA16:1 | 822.8 | 551.8 |
| TAG(48:1)_FA18:1 | 822.8 | 523.8 |
| TAG(48:2)_FA16:0 | 820.8 | 547.8 |
| TAG(48:2)_FA16:1 | 820.8 | 549.8 |
| TAG(48:2)_FA18:1 | 820.8 | 521.8 |
| TAG(48:2)_FA18:2 | 820.8 | 523.8 |
| TAG(50:0)_FA16:0 | 852.8 | 579.8 |
| TAG(50:0)_FA18:0 | 852.8 | 551.8 |
| TAG(50:1)_FA16:0 | 850.8 | 577.8 |
| TAG(50:1)_FA16:1 | 850.8 | 579.8 |
| TAG(50:1)_FA18:0 | 850.8 | 551.8 |
| TAG(50:1)_FA18:1 | 850.8 | 553.8 |
| TAG(50:2)_FA16:0 | 850.8 | 577.8 |
| TAG(50:2)_FA16:1 | 850.8 | 579.8 |
| TAG(50:2)_FA18:1 | 850.8 | 551.8 |
| TAG(50:2)_FA18:2 | 850.8 | 553.8 |
| TAG(50:3)_FA16:0 | 848.8 | 575.8 |
| TAG(50:3)_FA16:1 | 848.8 | 577.8 |
| TAG(50:3)_FA18:1 | 848.8 | 549.8 |
| TAG(50:3)_FA18:2 | 848.8 | 551.8 |

**Supplementary table 2: List of target genes included in the study and their respective names and functional category.**

| Gene | Gene name | Gene category |
| --- | --- | --- |
| *CPT1B* | Carnitine Palmitoyltransferase 1B | Beta-oxidation |
| *CPT2* | Carnitine Palmitoyltransferase 2 |  |
| *BMP15* | Bone Morphogenetic Protein 15 | Control of gene expression / epigenetics |
| *PAF1* | RNA Polymerase II Associated Factor |  |
| *REST* | RE1-Silencing Transcription factor |  |
| *STAT3* | Signal Transducer and Activator of Transcription 3 |  |
| *Dnmt1* | DNA (Cytosine-5-)-Methyltransferase 1 |  |
| *Dnmt3A* | DNA (Cytosine-5-)-Methyltransferase 3A |  |
| *Dnmt3B* | DNA (Cytosine-5-)-Methyltransferase 3B |  |
| *H3F3A* | H3 histone family member 3A |  |
| *H3F3B* | H3 histone family member 3B |  |
| *HDAC2* | Histone Deacetylase 2 |  |
| *TFAM* | Mitochondrial transcription factor A. |  |
| *ATF4* | Activating transcription factor 4 | Control of gene expression / stress response |
| *DDIT3* | DNA Damage Inducible Transcript 3 |  |
| *KEAP1* | Kelch-like ECH-associated protein 1 |  |
| *CDX2* | Caudal type homeobox 2 | Cell differentiation |
| *NANOG* | Nanog homeobox |  |
| *POU5F1* | POU class 5 homeobox 1 |  |
| *IGF1R* | Insulin-Like Growth Factor 1 Receptor | Cell growth |
| *IGFBP2* | Insulin-Like Growth Factor Binding Protein 2 |  |
| *IGFBP4* | Insulin-Like Growth Factor Binding Protein 4 |  |
| *ACTB* | Actin. beta | Housekeeping |
| *PPIA* | Peptidylprolyl Isomerase A |  |
| *ACACA* | Acetyl-CoA Carboxylase Alpha | Lipid metabolism |
| *ACSL1* | Acyl-CoA synthetase long-chain family member 1 |  |
| *ACSL3* | Acyl-CoA synthetase long-chain family member 3 |  |
| *ACSL6* | Acyl-CoA synthetase long-chain family member 6 |  |
| *EGFR* | epidermal growth fator receptor |  |
| *ELOVL6* | Fatty acid elongase 6 |  |
| *FADS2* | Fatty acid desaturase 2 |  |
| *FASN* | Fatty acid synthase |  |
| *GPAM* | Glycerol-3-Phosphate Acyltransferase |  |
| *HMGCS1* | 3-Hydroxy-3- Methylglutaryl-CoA Synthase 1 |  |
| *LIPE* | Lipase E |  |
| *PPARA* | peroxisome proliferator activated receptor alpha |  |
| *PPARG* | peroxisome proliferator activated receptor gama |  |
| *PPARGC1A* | Peroxisome proliferator-activated receptor gamma coactivator 1-alpha |  |
| *SCD* | Stearoyl-CoA desaturase |  |
| *SREBF1* | Sterol Regulatory Element Binding Transcription F1 |  |
| *ACAT1* | Acetyl-CoA acetyltransferase. mitochondrial | Energy metabolism / glycolysis |
| *ATP5L* | ATP Synthase |  |
| *MTIF3* | Melanogenesis Associated Transcription Factor |  |
| *NDUFA1* | NADH dehydrogenase [ubiquinone] 1 alpha |  |
| *PFKP* | Phosphofructokinase |  |
| *SDHA* | Succinate Dehydrogenase Complex Flavoprotein Subunit A |  |
| *SLC2A1* | Solute Carrier Family 2 Member 1 |  |
| *SLC2A3* | Solute Carrier Family 2 Member 3 |  |
| *SLC2A4* | Solute Carrier Family 2 Member 4 |  |
| *SLC2A5* | Solute Carrier Family 2 Member 5 |  |
| *G6PD* | Glucose 6 phosphate dehydrogenase |  |
| *GAPDH* | glyceraldehyde-3-Phosphate dehydrogenase |  |
| *PGK1* | Phosphoglycerate Kinase 1 |  |
| *BAX* | BCL2-Associated X Protein | Cell death |
| *BID* | BH3 interacting-domain death agonist |  |
| *CASP3* | Caspase 3. apoptosis-related cysteine peptidase |  |
| *CASP9* | Caspase 9. apoptosis-related cysteine peptidase |  |
| *ADCY3* | Adenylate Cyclase 3 | Other cell functions |
| *ADCY6* | Adenylate Cyclase 6 |  |
| *ADCY9* | Adenylate Cyclase 9 |  |
| *AQP3* | Aquaporin 3 |  |
| *CDH1* | cadherin 1 |  |
| *GFPT2* | Glutamine-fructose-6-phosphate transaminase 2 |  |
| *GSK3A* | Glycogen Synthase Kinase 3a |  |
| *HPRT1* | hypoxanthine phosphoribosyltransferase 1 |  |
| *NFKB2* | Nuclear Factor of Kappa Light Polypeptide Gene Enhancer in B-Cells 2 |  |
| *RGS2* | Regulator of G-protein signaling 2 |  |
| *MORF4L2* | Mortality factor 4 like 2 | Stress response |
| *XBP1* | X-Box Binding Protein 1 |  |
| *AKR1B1* | Aldo-Keto Reductase Family 1. Member B1 | Response to oxidative stress |
| *CAT* | Catalase |  |
| *GPX1* | Glutathione Peroxidase 1 |  |
| *GPX4* | Glutathione Peroxidase 4 |  |
| *HIF1A* | Hypoxia Inducible Factor 1 |  |
| *HMOX1* | Heme oxygenase |  |
| *HSF1* | Heat Shock Transcription Factor 1 |  |
| *HSP90AA1* | Heat Shock Protein 90 Alpha Family Class A Member 1 |  |
| *HSPA1A* | Heat Shock Protein A |  |
| *NFE2L2* | Nuclear factor (erythroid-derived 2)-like 2 |  |
| *NOS2* | Nitric Oxide Synthase 2 |  |
| *PRDX1* | Peroxiredoxin-1 |  |
| *PRDX3* | Peroxiredoxin-3 |  |
| *SOD1* | Superoxide dismutase 1. soluble |  |
| *SOD2* | Superoxide dismutase 2. mitochondrial |  |

**Supplementary table 3:** Summary of descriptive results for all 12 groups.

|  | **Pyruvate** ^&^  (µM) | **Lactate** ^&^  (µM) | **Mitochondrial activity** ^δ^  (arbitrary units) | **ROS** ^δ^  (arbitrary units) | **ATP** ^&^  (µM) | **Total FFA**^*^  (arbitrary units) | **C16:0**^*^  (arbitrary units) | **C18:0**^*^  (arbitrary units) | **Total TAG**^*^  (arbitrary units) | **Total**  **Chol ester** ^δ^  (arbitrary units) | **Blastocyst**  **rates**  (%) |
| --- | --- | --- | --- | --- | --- | --- | --- | --- | --- | --- | --- |
| **0F20** | 308.8 ±  8.12 | 5.358 ±  0.459 | 6152.06 ±  617.80 | 7013.70 ±  414.80 | 0.139 ±  0.015 | 0.217 ±  0.034 | 0.015 ±  0.002 | 0.027 ±  0.004 | 0.122 ±  0.015 | 0.040 ±  0.005 | 41.14 ±  5.88 |
| **0S20** | 339.5 ±  5.07 | 7.823 ±  0.324 | 6304.78 ±  1718.00 | 8437.69 ±  763.70 | 0.151 ±  0.026 | 0.300 ±  0.014 | 0.013 ±  0.003 | 0.028 ±  0.007 | 0.116 ±  0.004 | 0.046 ±  0.001 | 25.14 ±  3.75 |
| **2F20** | 322.9 ±  16.58 | 6.616 ±  0.605 | 10176.83 ±  887.80 | 8864.13 ±  432.60 | 0.279 ±  0.034 | 0.349 ±  0.014 | 0.016 ±  0.003 | 0.034 ±  0.006 | 0.127 ±  0.003 | 0.048 ±  0.002 | 38.65 ±  7.00 |
| **2S20** | 332.2 ±  8.81 | 6.775 ±  0.587 | 5379.57 ±  815.40 | 6916.16 ±  367.90 | 0.230 ±  0.026 | 0.330 ±  0.025 | 0.019 ±  0.004 | 0.042 ±  0.008 | 0.134 ±  0.004 | 0.054 ±  0.002 | 16.11 ±  3.43 |
| **5F20** | 304.9 ±  14.80 | 6.206 ±  0.488 | 4529.06 ±  477.70 | 8111.66 ±  690.20 | 0.159 ±  0.021 | 0.341 ±  0.005 | 0.018 ±  0.003 | 0.040 ±  0.008 | 0.132 ±  0.002 | 0.050 ±  0.001 | 35.33 ±  5.37 |
| **5S20** | 333.8 ±  17.32 | 7.072 ±  0.274 | 5101.12 ±  1088.00 | 6793.20 ±  486.10 | 0.174 ±  0.039 | 0.309 ±  0.039 | 0.022 ±  0.005 | 0.044 ±  0.010 | 0.144 ±  0.014 | 0.055 ±  0.004 | 14.4 ±  1.56 |
| **0F5** | 309.4 ±  18.48 | 6.365 ±  0.255 | 9173.75 ±  1493.00 | 5159.96 ±  705.60 | 0.221 ±  0.042 | 0.189 ±  0.015 | 0.035 ±  0.005 | 0.072 ±  0.011 | 0.157 ±  0.007 | 0.053 ±  0.003 | 45.6 ±  3.91 |
| **0S5** | 346.7 ±  20.32 | 8.561 ±  0.977 | 7855.07 ±  2478.00 | 6468.86 ±  640.90 | 0.172 ±  0.033 | 0.172 ±  0.030 | 0.045 ±  0.003 | 0.093 ±  0.007 | 0.189 ±  0.009 | 0.062 ±  0.003 | 22.64 ±  3.13 |
| **2F5** | 389.9 ±  18.93 | 7.378 ±  0.146 | 4334.05 ±  654.20 | 7430.34 ±  386.30 | 0.328 ±  0.064 | 0.191 ±  0.017 | 0.042 ±  0.005 | 0.083 ±  0.010 | 0.161 ±  0.005 | 0.059 ±  0.002 | 45.69 ±  4.28 |
| **2S5** | 376.4 ±  24.21 | 7.189 ±  0.620 | 4352.37 ±  772.70 | 4782.03 ±  642.40 | 0.114 ±  0.026 | 0.197 ±  0.022 | 0.040 ±  0.005 | 0.086 ±  0.007 | 0.156 ±  0.009 | 0.057 ±  0.003 | 30.19 ±  5.19 |
| **5F5** | 346.3 ±  24.38 | 6.919 ±  0.493 | 2573.90 ±  459.20 | 6578.96 ±  848.70 | 0.226 ±  0.043 | 0.181 ±  0.015 | 0.036 ±  0.003 | 0.068 ±  0.008 | 0.163 ±  0.007 | 0.053 ±  0.004 | 41.97 ±  4.79 |
| **5S5** | 363.8 ±  16.55 | 6.860 ±  0.397 | 2628.30 ±  839.70 | 5031.57 ±  790.30 | 0.244 ±  0.020 | 0.202 ±  0.027 | 0.022 ±  0.008 | 0.041 ±  0.015 | 0.156 ±  0.013 | 0.052 ±  0.003 | 33.04 ±  2.87 |

All values are mean ± S.E.M.

^&^ Absolute values for each sample were obtained by fluorimetry assays with the aid of a standard curve.

^δ^ Relative values for each embryo were obtained after background subtraction and normalized by the area of the embryo.

* Relative intensity of each ion was calculated as a function of the total count of ions in each group.

**Supplementary Table 4:** cleavage rates calculated for all groups.

|  | 20% O_2_ | 5% O_2_ | p-value |
| --- | --- | --- | --- |
| Fast cleavage (%)  (mean ± S.E.M) | 33.6 ± 2.51 | 37.4 ± 2.29 | 0.272 |
| Slow cleavage (%)  (mean ± S.E.M) | 28.5 ± 1.52 | 26.0 ± 1.54 | 0.271 |
| Late cleavage (%)  (mean ± S.E.M) | 6.1% ± 0.76 | 5.3 ± 0.67 | 0.411 |
| Non-cleaved (%)  (mean ± S.E.M) | 31.8% ± 1.7 | 31.3 ± 1.45 | 0.819 |
| Total Cleavage | 68.2% | 68.7% | - |

Values are considered statistically significant when p<0.05.

**Supplementary table 5:** Detailed information on the assignment of the scores according to the distribution on the quartiles.

| Variable | Raw values ** |  | Assigned Score |
| --- | --- | --- | --- |
| Pyruvate consumption ^δ^ | 255.9 – 316.8 (µM) | High | 0 |
|  | 316.8 – 361.9 (µM) | Intermediate | 1 |
|  | 361.9 – 469.4 (µM) | Low | 2 |
| Mitochondrial activity | 569.66 – 2780.05 (A.U.) | Low | 0 |
|  | 2780.05 – 4950.95 (A.U.) | Intermediate | 1 |
|  | 4950.95 – 8520.75 (A.U.) | High | 2 |
|  | 8520.75 – 17327.76 (A.U.) | Very High | 0 |
| ROS production | 13281.80 – 8529.83 (A.U.) | High | 0 |
|  | 8529.83 – 5486.98 (A.U.) | Intermediate | 1 |
|  | 5486.98 – 1539.36 (A.U.) | Low | 2 |
| ATP Production | 0.076 – 0.145 (µM) | Low | 0 |
|  | 0.145 – 0.253 (µM) | Intermediate | 1 |
|  | 0.253 – 0.512 (µM) | High | 2 |
| Lipid Ratio  FFA/ (TAG + Cholesterol) | 3.36 – 2.25 (A.U.) | High | 0 |
|  | 2.25 – 1.42 (A.U.) | Intermediate | 1 |
|  | 1.42 – 1.05 (A.U.) | Low | 2 |
| Blastocyst rate | 8.3 -20.0 (%) | Low | 0 |
|  | 20.0 – 47.9 (%) | Intermediate | 1 |
|  | 47.9 – 62.5 (%) | High | 2 |

** The authors advise for a careful interpretation of these values as most of the units are arbitrary and can vary according to the equipment used to measure fluorescency/luminescence. We do not intend to set thresholds, but rather show the spectrum of variation for each parameter.

^δ^ Raw pyruvate values measured in culture media are inversely proportional to consumption (e.g. higher values indicate lower consumption).
